# Low but highly geographically structured genomic diversity of East Asian Eurasian otters and its conservation implications

**DOI:** 10.1111/eva.13630

**Published:** 2023-12-23

**Authors:** Shou‐Hsien Li, Chia‐fen Yeh, Nian‐Hong Jang‐Liaw, Shih‐Wei Chang, Yu‐Hsiu Lin, Cheng‐En Tsai, Chi‐Cheng Chiu, Chien‐Wen Chen, Hui‐Ru Ke, Qiaoyun Wang, Yiwei Lu, Kaidan Zheng, Pengfei Fan, Lu Zhang, Yang Liu

**Affiliations:** ^1^ School of Life Science National Taiwan Normal University Taipei Taiwan; ^2^ Conservation and Research Center Taipei Zoo, Taipei Taiwan; ^3^ Division of Zoology Endemic Species Research Institute Nantou Taiwan; ^4^ Genomics BioSci & Tech Co., Ltd. New Taipei City Taiwan; ^5^ State Key Laboratory of Biocontrol, School of Ecology/School of Life Sciences Sun Yat‐Sen University Guangzhou China; ^6^ Zhejiang Museum of Natural History Zhejiang Biodiversity Research Center Hangzhou China

**Keywords:** conservation genomic, *Lutra lutra*, population genetics – empirical, wildlife management

## Abstract

Populations of Eurasian otters *Lutra lutra*, one of the most widely distributed apex predators in Eurasia, have been depleted mainly since the 1950s. However, a lack of information about their genomic diversity and how they are organized geographically in East Asia severely impedes our ability to monitor and conserve them in particular management units. Here, we re‐sequenced and analyzed 20 otter genomes spanning continental East Asia, including a population at Kinmen, a small island off the Fujian coast, China. The otters form three genetic clusters (one of *L. l. lutra* in the north and two of *L. l. chinensis* in the south), which have diverged in the Holocene. These three clusters should be recognized as three conservation management units to monitor and manage independently. The heterozygosity of the East Asian otters is as low as that of the threatened carnivores sequenced. Historical effective population size trajectories inferred from genomic variations suggest that their low genomic diversity could be partially attributed to changes in the climate since the mid‐Pleistocene and anthropogenic intervention since the Holocene. However, no evidence of genetic erosion, mutation load, or high level of inbreeding was detected in the presumably isolated Kinmen Island population. Any future in situ conservation efforts should consider this information for the conservation management units.

## 1 INTRODUCTION

Biodiversity, which is vital to both ecosystem function and human well‐being (Naeem et al., 2016), is under threat globally. Among the three major realms of ecosystems, global freshwater biodiversity is declining at unprecedented rates (Brondízio et al., 2019). Taking freshwater mammals as an example, 40% of all species are currently listed as threatened species (Sanders et al., 2023), and some larger freshwater mammals, such as Baiji dolphin *Lipotes vexillifer* decreased dramatically and may have been extinct (Turvey et al., 2007). To prevent further biodiversity loss, it is critical to formulate appropriate strategies to conserve them in individual conservation management units (MUs). An MU is a population whose growth rate mainly depends on its local birth and death rates rather than gene flow from other such units (Moritz, 1994). Due to their demographic independence, each MU has to be monitored and managed individually.

**FIGURE 1:**
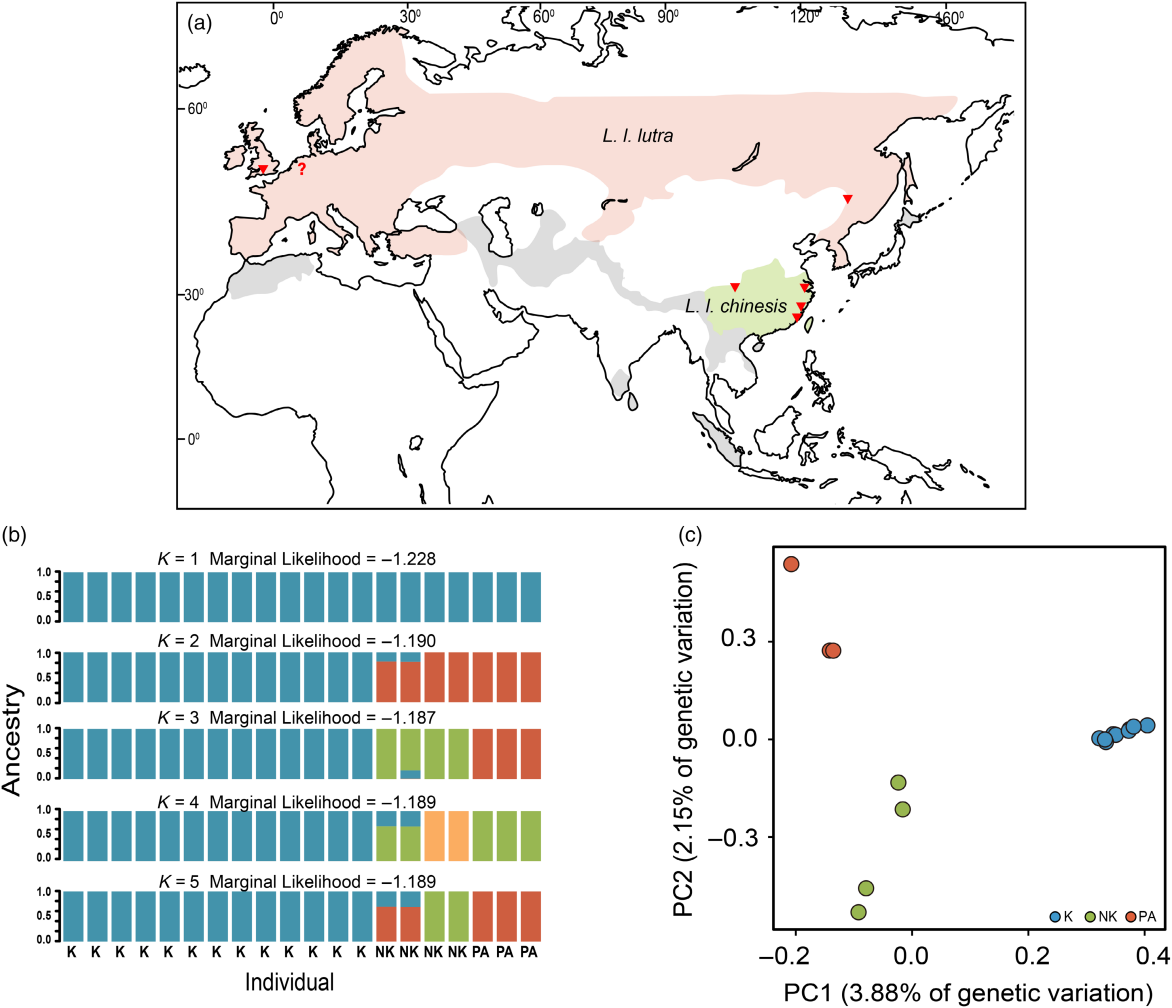
(a) Sampling localities of Eurasian otters; “?” denotes the individual from Taipei Zoo originating in Germany; Orange: range of nominate subspecies *L. l. lutra*; green: range of subspecies *L. l. chinensis*; grey: range of other subspecies unsampled in the current study; (b) Results of fastSTRUCTURE indicate that *k* = 3 has the highest likelihood; K denoted individuals collected from Kinmen Island, NK denoted individuals of *L. l. chinensis* collected from Sichuan, Northern Fujian, and Zhejiang. (c) Plot of PC1 vs. PC2 of each genome. The range map of Eurasian otters is modified from Hung and Law (2016).

As one of the apex predators of freshwater ecosystems, the Eurasian otter (*Lutra lutra*) occurs throughout Eurasia and parts of northern Africa (Hung & Law, 2016; Figure 1a). Due to anthropogenic intervention, their numbers have declined drastically across most of their range since the 1950s (Roos et al., 2021). The species is listed as ‘Near Threatened’ in the Red List of Threatened Species (IUCN, 2021). In Europe, efforts at standardized surveying and monitoring have indicated that some local populations are gradually recovering from the brink of extinction (such as in Great Britain; Mason & Macdonald, 2004). Reintroduction programs have also successfully re‐established some local populations (e.g., Balestrieri et al., 2021). The sparse information available about East Asian otters indicates that they have also sharply declined in number since the 1950s (Conroy et al., 1998). Various human interventions have sequestered Eurasian otters into highly fragmented populations in most of its East Asian range (Conroy et al., 1998). In China, they can currently only be found in the Qinghai‐Tibetan Plateau, North‐east China, and peripheral islands along the south‐eastern coastal region (Zhang et al., 2018). However, the Eurasian otter population in the southern Korean Peninsular has recovered significantly (Jo et al., 2017, 2020), some local populations, such as that in Taiwan (Lee, 1997), have been considered to be extinct. There has been no reintroduction program for Eurasian otters in Asia yet.

Information about genetic variation among the Eurasian otters of East Asia greatly helps our efforts to construct appropriate MUs for their conservation. Mitochondrial (mt) DNA analysis results suggested that this species diverged little across its entire range (Waku et al., 2016). However, this could have been driven by mechanisms such as selection (Ballard & Whitlock, 2004), introgression (McGuire et al., 2007), or the presence of nuclear mitochondrial pseudogenes (Bensasson et al., 2001). Demographic events, such as a population expansion after a severe historical demographic bottleneck, could also have led to low genetic diversity and divergence (Ellegren & Galtier, 2016). The microsatellite‐based analysis also suggested that the genetic diversity of the European population of Eurasian otters was low (reviewed by Hung & Law, 2016). However, the genomic diversity of the East Asian population is unknown, and the need to close this knowledge gap is urgent.

Human intervention has driven otter populations in East Asia to become as small and isolated as the Kinmen Island population (Hung et al., 2004). Kinmen is a small island (151.7 km^2^) off the Fujian coast of China, where fecal DNA typing identified fewer than 100 individuals in each recent year (Jang‐Liaw, 2021). No otters have been recorded recently in the nearby mainland region (Zhang et al., 2018), so the Kinmen otters have long been considered an isolated relict population (Lee, 1996). Small isolated populations can significantly reduce effective population size (*N*_e_), resulting in high genetic drift and inbreeding levels. This could lead to a reduction in both genetic diversity (genetic erosion; Allendorf, 1986) and the efficacy of purifying selection in removing deleterious mutations from a population (Kirkpatrick & Jarne, 2000) such that mildly deleterious mutations accumulate (mutation load; Kimura et al., 1963). Expression of recessive deleterious mutations would also manifest through inbreeding in small populations (inbreeding load; Charlesworth & Charlesworth, 1987). All of these factors could compromise the fitness of a population and its evolutionary potential. Genetic problems for a small population can be mitigated by bringing in new genetic variations through population supplementation or genetic rescue (Bell et al., 2019). To practice genetic rescue, levels of inbreeding and inbreeding depression in the targeted population need to be verified, and individuals from a source population in the same MU or close phylogenetic relative population should be preferred (Hedrick & Fredrickson, 2010) to avoid unwanted outbreeding depression (Lynch, 1991). Consequently, information on MUs is a prerequisite for incorporating genetic rescue in conservation practices.

Here, we re‐sequenced whole‐genomes from Eurasian otters collected from three East Asian populations to address the pattern of their genetic diversity at two geographic levels. First, we assayed their genomic diversity, inferred how they are geographically structured at the regional scale, and reconstructed their historical demographic trajectories to understand how their genetic diversity was shaped by historical events (Ellegren & Galtier, 2016). Second, to evaluate the genetic consequences of their recent population fragmentation, we assessed the levels of genetic erosion, inbreeding, and mutation load in the small, isolated Kinmen Island population at the local scale. The results of this study will significantly enhance our understanding of how the genetic diversity pattern of Eurasian otters was shaped in East Asia and offer information to delineate the population into management units. It also provides the first glimpse of how recent human intervention affects the genetic health of the otter. All of these results could be important in constructing conservation plans.

## 2 MATERIALS AND METHODS

### 2.1 Population sampling and whole genome resequencing

We re‐sequenced entire genomes of eighteen Eurasian otters from the eastern Qinghai‐Tibetan plateau (Qingchuan, 104.8° E, 32.5° N, *N* = 2), north‐east (Tahe, 124.7° E, 52.3° N, *N* = 1), and south‐eastern coastal regions (Xiangshan, 122.0° E, 29.3° N, *N* = 1; Fuding, 120.2° E, 27.4° N, *N* = 1; Kinmen, 118.3° E, 24.4° N, *N* = 13) of China (Figure 1), which had been killed in accidents. Of these regions, otters are relatively abundant in the eastern Qinghai‐Tibetan plateau and northeast China (Han & Shi, 2019). We also sequenced a German individual kept in Taipei Zoo, which served as the outgroup and the published annotated draft genome assembly of a male Eurasian otter individual collected in Wincanton, Somerset, UK (BioProject accession number PRJEB35340, https://www.ncbi.nlm.nih.gov/bioproject/?term=PRJEB35340; Mead et al., 2020). The reference genome is 2.44 gigabases (Gb) in span and comprises 20 pseudochromosomes.

Gross genomic DNA was extracted from tissue samples following a protocol from Gemmell and Akiyama (1996) except to dissolve the DNA pellet in distilled water for later use. A whole‐genome library with an insert size of 350 bp was constructed for each individual with a KAPA HyperPlus kit (Illumina). The sequencing data were generated by Genomics Biotech (Taipei) using an Illumina Novaseq 6000 platform.

### 2.2 Re‐sequence mapping and variant calling

We used fastp 0.21 (Chen et al., 2018) to trim adaptors and poor sequence quality (<Q30) from raw reads. We removed reads if more than 40% of bases with lower than Q30 and reads of <15 bp lengths. The average quality score for trimmed reads was 36. We used the algorithm *BWA‐MEM* in BWA 0.7.13 (Li & Durbin, 2010) to align all trimmed reads from each individual to the reference genome. The software *Samtools* v1.3.1 (Li, 2011) *fixmate* command was used for mate coordinates, *f3F268 –q30* for discard mapping quality (MAPQ) lower than 30 and remove unpaired reads, then tagged duplicated reads by the command *markdup* of *Samtools* that would be excluded from the subsequent analysis. Then, we used the command *Genomecov* of bedtools (Li, 2011) to calculate the average coverage of each individual. The average coverage of the Eurasian otters was 47.38× (SE = 1.86; Table S1). We used bcftools mpileup and bcftools call (Li, 2011) to perform variant calling. Then, we used bcftools view (‐m2 ‐M2 ‐v snps) to only view biallelic SNPs in the subsequent analysis. We used vcftools (‐remove ‐indel) to remove indels from the VCF file. We retained biallelic SNP sites with FMT/DP (coverage) > 10, FMT/GQ (genotype quality score) > 20, and no missing individual. Totally 10,029,537 SNPs were identified. Among them, 9,962,859 were autosomal SNPs.

### 2.3 Geographic organization of genomic diversity

To investigate the geographic structure of otters in East Asia, we used *PLINK 1.9* (Purcell et al., 2007) to filter out autosomal SNPs with *r* ^2^ ≥ 0.2 (20 Kb window size with 1 Kb step sliding window), which significantly deviated from the Hardy–Weinberg expectation (*p* < 0.1) with minor allele frequency <0.10. In total, 216,388 unlinked autosomal SNPs were retained. Then, we used fastSTRUCTURE version 1.0 (Raj et al., 2014) to infer the most likely number of ancestral clusters from these unlinked autosomal SNPs. The prior of ancestral clusters (*K*) was set to 1–5 with 10 replicate runs for each *K* value. We used the *chooseK*.*phy* script (Raj et al., 2014) to determine the best model of the value *K*.

We used principal components analysis (PCA) with *PLINK 1.9* to analyze the unlinked autosomal SNP set. We plotted the score of the first component (PC1) against that of the second component (PC2) to depict the genetic similarity of different genomes.

Then, we calculated the mean heterozygosity (*H*, number of heterozygous SNPs/lengths of the reference genome) for the autosomes of each group as a measurement of genomic diversity. We compared the heterozygosity of each otter group with those of other carnivorous mammals (Brüniche‐Olsen et al., 2018) to evaluate their levels of genomic diversity. We also used the equation in Smith and Kronforst (2013) to calculate the genetic distance, *d*_xy_, between each pair of populations (Nei, 1987; Nei & Li, 1979) with frequency data. The net genetic distance (*d*_a_; Nei & Li, 1979), *d*_xy_ subtracted from the mean nucleotide diversity of any two populations, was used to estimate the nucleotide difference accumulated since the two populations split.

### 2.4 Historical demography.

We applied three model‐free methods, namely SMC++ (Terhorst et al., 2017), Stairway Plot 2 (Liu & Fu, 2020), and GONE (Santiago et al., 2020), to infer historical demographic trajectories in different time scales based on 9,962,859 autosomal SNPs. The first two programs employ the coalescent‐based method, utilizing information on the site frequency spectrum (SFS) to infer *N*_e_ of the population through time. We used the script easySFS (https://github.com/isaacovercast/easySFS) to generate the site frequency spectrum for each population. Only individuals in the same ancestry group of the fastSTRUCTURE analysis were considered population members for the following demographic analyses. SMC++ allows us to estimate the divergence time between populations with a clean split model by assuming no gene flow occurs after the population split.

We used the linkage disequilibrium‐based method implemented in the software GONE (Santiago et al., 2020) to infer very recent population trajectories (<200 generations) for populations with sample sizes >10 (i.e., Kinmen population in this case). The data was set to the unknown phase. The number of generations was set to 200. The number of bins was set to 400. The maximum recombination rate between pairs of analyzed SNPs was set to 0.05 (hc = 0.05). The maximum approximate number of SNPs per chromosome to be analyzed was set to 50,000. The number of replicates was set to 40. We used the average recombination rate of dogs (0.97 cM/Mb; Wong et al., 2010) as a proxy for Eurasian otters. For all analyses, the generation time of the Eurasian otter was set to 7 years (Hauer et al., 2002), and the mutation rate was set to 1.05 × 10^−8^ per site per year (the mutation rate of wolves, Koch et al., 2019).

### 2.5 Level of inbreeding

We used *PLINK* v1.9 to calculate the number and length (>1000 Kb) of ROHs (Runs of homozygosity, long stretches of identical homologous genomic segments) in each autosomal genome based on 9,962,859 non‐LD filtered autosomal SNPs. We followed Brüniche‐Olsen et al. (2018) to set the parameters for ROH identification (*Plink* commends: ‐homozyg‐kb 1000 ‐homozyg‐snp 20 ‐homozyg‐window‐snp 20 ‐homozyg‐window‐missing 1 ‐homozyg‐window‐het 1 ‐homozyg‐window‐threshold 0.01). We calculated the sum of total ROH length (SROH), the number of ROH fragments (NROH), the average length of ROH fragments (LROH; SROH/NROH), and the genomic inbreeding coefficient, *F*_ROH_ (SROH/the effective autosomal genome size; Keller et al., 2011) for each genome sequenced. The level of *F*_ROH_ of each group was compared to those of other carnivorous mammals (Brüniche‐Olsen et al., 2018) in different IUCN Red List categories.

### 2.6 Accumulation of mutation load

We used *ANNOVAR* (Wang et al., 2010) to annotate substitutions in the coding region of a genome into three categories, synonymous, non‐synonymous, and loss‐of‐function (LoF, including stop codon gain and loss) substitutions. Due to the lack of transcriptome data, the other category of LoF mutations, such as splice deletion, was not included here. Because indels were excluded from the dataset, we cannot identify frameshift mutations when counting LoF per genome. We defined the allele identical to the homozygous allele in the outgroup, small coated otter *Lutrogale perspicillata* (NCBI Bioproject PRJNA841998; de Ferran et al., 2022), as the ancestral allele; others as the derived allele. However, if SNPs were heterozygotes or missing in the outgroup, their ancestral state would be classified as unclassified, and these SNPs were excluded from the subsequent analysis. Then, we counted the number of non‐synonymous and loss‐of‐function substitutions in each genome as accumulated potentially deleterious mutations or mutation load. We used the Grantham score (Grantham, 1974) to measure the potential phenotypic effect of substitutions. We followed Chun and Fay (2009) to classify Grantham scores into three categories: conservative changes (0–50), moderate changes (51–100), and radical changes (>100). Then, we assumed all moderate and extreme changes in amino acids to be potentially deleterious. Assuming different non‐synonymous substitutions to have an independent and additive effect, we summed up the Grantham score of substitutions categorized as moderate or radical changes for each genome to quantify the accumulated potential phenotypic effect.

### 2.7 Statistical analysis

We used ANOVA with the Tukey–Kramer HSD test to compare whether levels of genetic diversity (heterozygosity), inbreeding, and mutation load were equal among the defined conservation management units. All these tests were performed with the software JMP 7.0.

## 3 RESULTS

### 3.1 Genetic structure and genomic diversity of the Eurasian otter

The results of fastSTRUCTURE suggest that the optimal model of the Eurasian otter individuals sampled comprises three ancestral clusters (*K* = 3, marginal likelihood = −1.187; Figure 1b). The two European individuals are clustered into the same ancestral group as the sample from North‐east China (Palearctic group). All the Kinmen individuals derive from the same ancestry (Kinmen group). Two inland (Qinghai‐Tibetan) samples and two coastal samples (sampled from northern Fujian and Zhejiang) share the same ancestry (non‐Kinmen group). All individuals were assigned to a single ancestry with high ancestry coefficients (>0.9). The results of the PCA analysis (Figure 1c) are consistent with that of the fastSTRUCTURE analysis. The first principal component (3.88% of variance) separates the Kinmen individuals (PC1 > 0.2) from the other samples (PC1 < 0.0); PC2 (2.15% of variance) further divides individuals from locations other than Kinmen into two clusters, the European and North‐east China samples (PC2 < 0.0) and the non‐Kinmen East Asian individuals (PC2 < −0.1). Therefore, our data support subdividing East Asian Eurasian otters into at least three clusters: Palearctic (northern China and Europe), non‐Kinmen (Qinghai‐Tibetan with northern Fujian and Zhejiang), and Kinmen (the Kinmen Island). We found that the level of heterozygosity (mean ± SE)is similar for all three otter groups (*F* = 2.0024, *p* = 0.166); it is 0.71±0.04, 0.91 ± 0.07, and 0.82 ± 0.08 × 10^−3^ for the Palearctic, non‐Kinmen, and Kinmen groups, respectively (Table S2).

We observed that the genome‐wide genetic divergence between the three populations is very shallow: *d*_xy_ was estimated to be 0.0011 between the Palearctic group and the other two East Asian groups and 0.0011 between the Kinmen and non‐Kinmen groups. The net genetic distance, *d*_a_, was estimated to be 0.00033 between the Palearctic and Kinmen groups and 0.00022 between the non‐Kinmen and the Palearctic or Kinmen groups, respectively. Compared with other carnivore genomes (Brüniche‐Olsen et al., 2018), the mean heterozygosity $\bar{H}$ of the three groups of Eurasian otter genomes are low and similar to those of the threatened (critically endangered+ endangered+ threatened species in the IUCN Red List of threatened species) species ($\bar{H}$ = 0.0010 ± 0.0010; *N* = 8), but lower than those of the un‐threatened (nearly threatened+ least concern species in the IUCN Red List of threatened species category) species ($\bar{H}$ = 0.0038 ± 0.00075; *N* = 15) (Figure 3a; Figure S1).

### 3.2 Historical demographic trajectories of the Eurasian Otter

**FIGURE 2:**
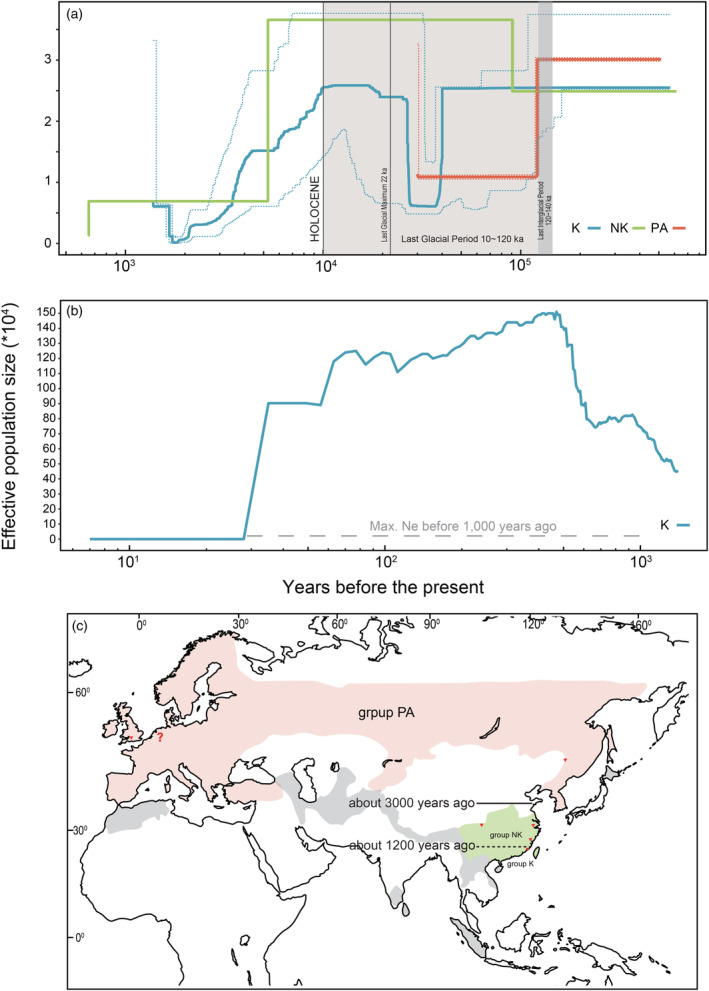
Historical demographics of Eurasian otters. (a) Results of Stairway Plot 2 analysis for the three groups; (b) Results of GONE analysis for the Kinmen group; the dashline (*N*_e_ = 40,000) indicates the maximum *N*_e_ of the Kinmen group before 1000 years ago. (c) It is estimated by Stairway Plot 2 in Figure 1a: The divergence time between three phylogroups of Eurasian otters in East Asia. The divergence data were inferred from the results of SMC++ (see Figure S2).

Stairway Plot 2 analysis (Figure 2a) suggested that *N*_e_ of the Kinmen group was about 25,499 since the mid‐Pleistocene (about 0.5 million years ago, Mya) to the onset of Holocene (about 10,000 years ago), except there had a drastic decline (*N*_e_ drop to <12,908) during the last glacial period (40,000–27,000 years ago). Then, it gradually declined from more than 25,864 to 1158 in the late Holocene (approximately 1700 years ago). Then, it recovered to more than 6727 recently (1380 years ago). Results of SMC++ showed a similar trajectory (Figure S2a,c). Results of LD‐based GONE analysis (Figure 2b) suggest that starting from 1400 years (200 generations) ago, *N*_e_ of the Kinmen population, starting from approximately 4.5 × 10^5^, reached spuriously high (>1.5 × 10^6^; see ‘Section 4’) from 1.4 × 10^3^ to 30 years ago; finally, it dropped to <127 at four generations ago (28 years; *N*_e_: 32.6–127.2).

Results of the Stairway Plot 2 analysis (Figure 2a) indicate that the historical *N*_e_ of the Palearctic group started from about 30,114 at the mid‐Pleistocene (about 512,754 ago) and then dropped to approximately 22,838 within the last interglacial period (around 120,000–140,000 years ago). Then, it dropped drastically to 12,128 in the late Pleistocene (29,946–119,947 years ago). SMC++ produced a more recent trajectory for the Palearctic group (Figure S2a,b), complemented by the Stairway Plot 2: *N*_e_ started from about 15,090–18,452 at the time split from other groups, then decreased to 4059–4963, followed by a bounce to 26,023–31,818 recently. For the non‐Kinmen group, results of the Stairway Plot 2 (Figure 2a) suggest that its *N*_e_ was about 24,894 in the mid‐Pleistocene (around 0.6 mya). Then, it grew to more than 36,591 approximately 90,000 years ago. Starting about 5200 years ago, it gradually declined to 1303 about 650 years ago. Results of SMC++ produce a similar demographic trend for the non‐Kinmen group (Figure S2a,c). Due to our limited sample size, we only applied GONE to analyze the Kinmen group.

Assuming no gene flow after the population splits, the split time estimated by SMC++ consists of their current taxonomic treatment: the Palearctic group (*L l. lutra*) first split from the Kinmen group, *L. l. chinensis*, about 3000 years ago (Figure S2b). Then, it was followed by the division between the Palearctic and non‐Kinmen groups, the other *L. l. chinensis* group, around 2698 years ago (Figure S2a). The Kinmen and non‐Kinmen groups, two *L. l. chinensis* groups split in the late Holocene (Figure S2c) around 1178 years ago.

### 3.3 Low inbreeding coefficients for the East Asian Eurasian otter

**FIGURE 3:**
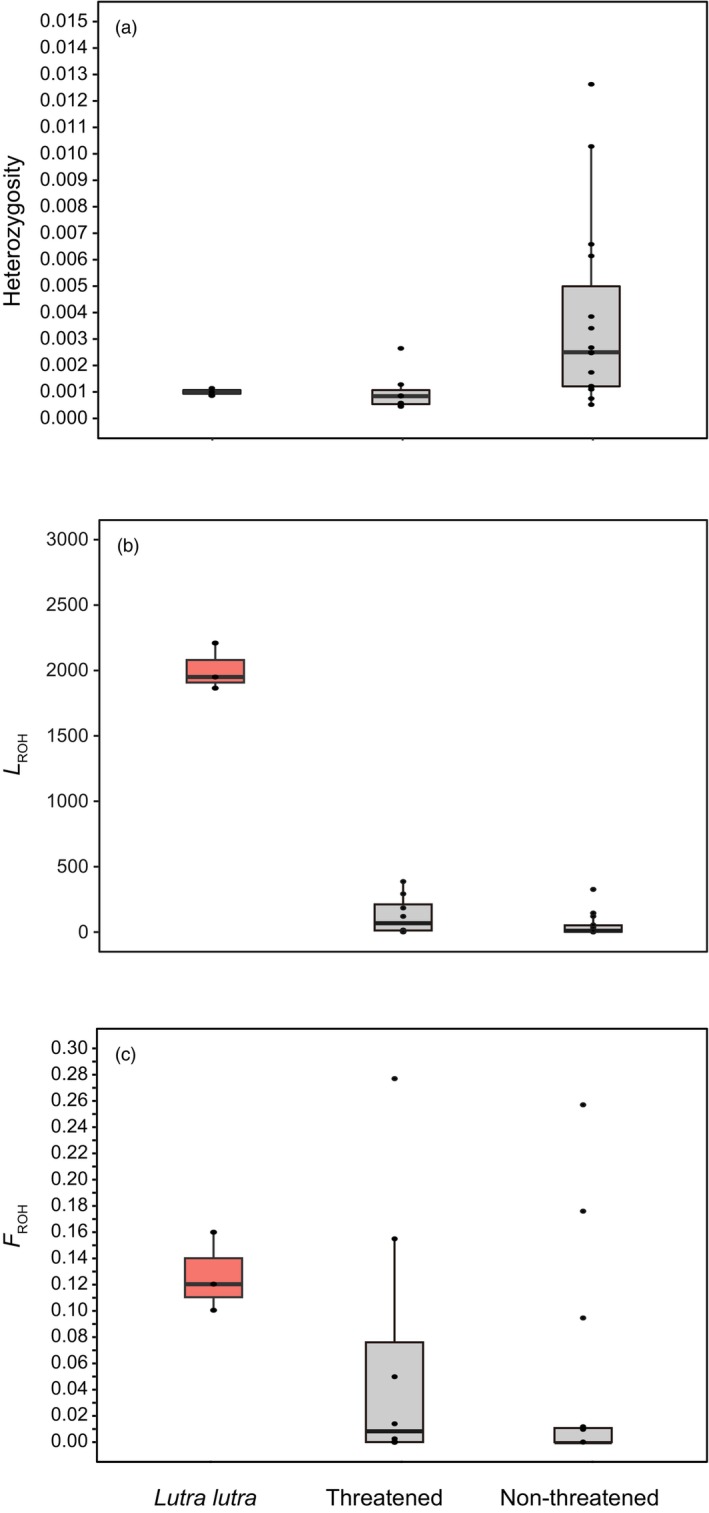
Comparing (a) the genetic diversity (heterozygosity), (b) Average length of ROHs (*L*_ROH_), and (c) Genomic inbreeding coefficient (*F*_ROH_) of the three groups of Eurasian otters with those of other published carnivorous mammals (Brüniche‐Olsen et al., 2018). Threatened species include species listed in the critically endangered+ endangered+ threatened species categories of the IUCN Red List of threatened species; the un‐threatened species include species listed in the nearly threatened+ least concern species of the IUCN Red List. Only *F*_ROH_ based on ROHs > 1000 Kb are presented here.

Our genomic data suggested that the case of close inbreeding (*F*_ROH_ > 0.1) could be detected in all three populations of East Asian European otters (Table S3; Figure 3). However, their levels of inbreeding do not significantly deviate from that of the threatened and non‐threatened carnivores (*F* = 1.4982, *p* > 0.05).

We found evidence to support recent inbreeding in all Eurasian otter groups (Figure 3). Among East Asian European otters, the genomic inbreeding coefficient, *F*_ROH_, is estimated to be 1.04 × 10^−1^ for the Kinmen Island population, which is smaller than that of the other two populations: 1.25 × 10^−1^ and 1.58 × 10^−1^ for the non‐Kinmen and Palearctic populations, respectively.

By further comparing the other two ROH statistics (*L*_ROH_ and *N*_ROH_) to the same statistics derived from published genomes of other carnivorous mammals (Brüniche‐Olsen et al., 2018), we found that Eurasian otters had significantly higher levels of *L*_ROH_ but not *N*_ROH_ (*N* = 15) (*F* = 347.044, *p* = 0.0001; and 0.2873 for *N*_ROH_, *p* > 0.05; Figure S1). Therefore, even the levels of *F*_ROH_ could be similar to the threatened and non‐threatened carnivore species. However, the long *L*_ROH_ suggested that the Eastern Asian European otter population might experience inbreeding more recently than other carnivores.

### 3.4 No higher mutation load in the Kinmen Island otters

**FIGURE 4:**
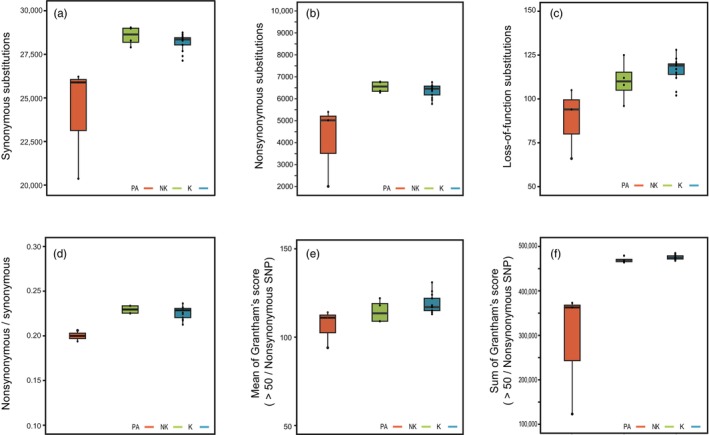
Numbers of (a) Synonymous, (b) Non‐synonymous, and (c) Loss‐of‐function (LoF) substitutions per genome for the three groups of Eurasian otters. (d) Mutational load, non‐synonymous/synonymous substitutions, (e) Average Grantham score per putatively deleterious non‐synonymous substitution, (f) Sum of the Grantham score for the putatively deleterious non‐synonymous substitution (Grantham score > 50) per individual. K, NK, and PA denote Kinmen, non‐Kinmen, and Palearctic groups.

Each Eurasian otter carries 27,627 synonymous, 6067 non‐synonymous, and 103.75 LoF substitutions (Table S4). However, we observed that the presumed relict Kinmen population has a lower mutation load (Figure 4) than theoretically expected. The Kinmen and non‐Kinmen groups have comparable levels of synonymous substitutions per genome (mean ± SE, hereafter, Kinmen group: 28,148.7 ± 333.9; non‐Kinmen group: 28,513.3 ± 601.9; Figure 4a), but the non‐Kinmen group has significantly more synonymous substitutions than the Palearctic group (24,182.3 ± 695.1; Tukey–Kramer HSD test, *p* < 0.05). The Kinmen and non‐Kinmen groups have similar levels of non‐synonymous substitutions per genome (Kinmen group: 6363.8 ± 192.1; non‐Kinmen group: 6544.3 ± 346,3), which are both significantly more than found in the Palearctic group (4142.3 ± 399.9; Tukey–Kramer HSD test, *p* < 0.05; Figure 4b). The Kinmen and non‐Kinmen groups also have similar levels of LoF substitutions per genome (Kinmen group: 116.5 ± 2.9; non‐Kinmen group: 110.2 ± 5.2), which are also significantly more than those found in the Palearctic group (88.3 ± 6.0; Tukey–Kramer HSD test, *p* < 0.05; Figure 4c). We calculated the ratio of non‐synonymous/synonymous substitutions as a proxy for the mutation load carried by each individual. We found that the Kinmen and non‐Kinmen groups have similar proportions of non‐synonymous/synonymous substitutions (Kinmen group: 0.225 ± 0.005; non‐Kinmen group: 0.229 ± 0.011), which are significantly higher than the ratio for the Palearctic group (0.166 ± 0.012; Tukey–Kramer HSD test, *p* < 0.05; Figure 4d). Individuals from the three populations have non‐equal Grantham scores (*F* = 18.66, *p* < 0.0001) (Grantham, 1974) for each non‐synonymous substitution they carried (mean ± SE: 80.1 ± 2.65; 89.1 ± 1.27, and 85.8 ± 2.3 for the Palearctic, Kinmen, and non‐Kinmen groups respectively) (Figure 4e). The mean Grantham score for non‐synonymous substitutions with a more significant phenotypic effect (Grantham score > 50; Chun & Fay, 2009) differs between the three groups (*F* = 4.78, *p* = 0.022; 106.5 ± 3.6; 118.9 ± 1.7 and 114.6 ± 3.1 for the Palearctic, Kinmen, and non‐Kinmen groups, respectively). The sum of Grantham scores of nonsynonymous sites with Grantham score > 50 is similar for the Kinmen and non‐Kinmen groups (Kinmen group: 475,425 ± 13,534; non‐Kinmen group: 469,461 ± 24,399), both of which are greater than that of the Palearctic group (286,365 ± 28,174, *F* = 18.88, *p* < 0.0001) (Figure 4f).

## 4 DISCUSSION

### 4.1 The Pleistocene climatic event and Holocene anthropogenic intervention drive the genetic organization of Eurasian otters in East Asia

Our results (Figure 1) indicate that the Palearctic group in our study corresponds to the *L. l. lutra* from Portugal to South Korea (Kruuk, 2006). Mitochondrial‐based phylogeny also suggested that samples from the Palearctic region, except the Korean Peninsula, form a shallow clade (du Plessis et al., 2023). A similar shallow and widespread lineage over northern Eurasia has also been found in organisms such as the grey wolf *Canis lupus* (Pilot et al., 2019), the great spotted woodpecker *Dendrocopos major* (Zink et al., 2002), and the mountain avens *Dryas octopetala* (Skrede et al., 2006). During the Pleistocene epoch, the ice sheet in the northern Palearctic extended from England to Siberia (Svendsen et al., 2004). The wide range of the single lineage in the north of the Eurasian continent could have been rendered by the rapid expansion from a single glacial refuge (Korsten et al., 2009). This supposition is supported by consistent demographic trajectories of all three otter groups around the early late Pleistocene (0.6 Mya, million years ago); this period corresponded to the mid‐Pleistocene transition (MPT, 0.7–1.25 million years ago) when glacial maxima had shifted from 41,000‐year cycles to 100,000‐year cycles and the last glacial period (Figure 3a). Before the end of the MPT (approximately 650,000 years ago), the most severe ice age of the late Pleistocene had occurred (Siegenthaler et al., 2005). It caused glaciers and deserts to expand to their maximum extent (Wu et al., 2002). Therefore, it might wipe out the ancestral populations of Eurasian otters from most of their extant range and push them into a single refuge.

Consisting with the mitochondrial DNA‐based phylogeny (du Plessis et al., 2023), our results support that southern and inland Chinese otters should be recognized as subspecies *L. l. chinensis* (Hung & Law, 2016). Results of SMC++ analysis (Figure S2a) suggest that these groups split from the Palearctic group around 3000 years ago. It is close to the 4.2‐kiloyear BP aridification event (4.2 K event), a long‐term drought in the Holocene. At that time, the vegetation of north China (30–40° N) would have been cool and dry steppe, which might have formed a habitat barrier between the mesic forest in north‐east China and Central China (Li et al., 2019; Ni et al., 2014). This arid belt has been considered the boundary between the Palearctic and Sino‐Japan Realms (Holt et al., 2013). Many pan‐Eurasian organisms have a phylogeographic divide in this arid belt. Examples include the Korean field mouse *Apodemus peninsulae* (Sakka et al., 2010), the azure‐winged magpie *Cyanopica cause* (Fok et al., 2002), and the temperate‐deciduous walnut tree, *Juglans mandshurica* (Bai et al., 2010, 2016).

Results of SMC++ (Figure S2c) suggest that the Kinmen and non‐Kinmen groups appear to have diverged roughly 1200 years ago (Figure S2c). Notwithstanding certain events like the 4.2 K event, the climate and vegetation of East Asia have remained relatively stable (Ni et al., 2014). This implies that the recent divergence of these groups may not be due to habitat barriers created by paleoclimatic events. A more plausible explanation could be human intervention.

Historically, the cradle of East Asian civilization was the Yellow River Valley in Northern China. This region is home to thousands of Neolithic archaeological sites (Li et al., 2009) and was the birthplace of all early Chinese dynasties. People from this region, often referred to as the Northern Han, migrated southward in three significant waves (Wen et al., 2004). Particularly during the second migration wave (AD 618–907), millions moved from the north to the south, leading to a substantial increase in the population density of southern China (Dong et al., 2022). This increase in population necessitated the transformation of the region's dominant mesic forests (Ni et al., 2014) into agricultural land, mainly rice paddies (Dong et al., 2022) and other anthropogenic habitats. Such vast habitat transformation would undeniably reduce and fragment the otter habitats, causing them to diminish from the fragmented habitat. Habitat reduction and fragmentation can reduce *N*_e_ and restrict gene flow between habitat patches, thereby promoting divergence (Jump & Peñuelas, 2006).

### 4.2 Human interventions since the Holocene might drastically reduce the genetic diversity of the Eurasian otter

Our historical demographic reconstruction suggests that *N*_e_ of the Eurasian otter populations in the Kinmen and the non‐Kinmen groups gradually declined in the Holocene (Figure 2). A similar *N*_e_ trend has also been found in green peafowl *Pavo muticus* (Dong et al., 2021) and six avian species in southern China (Dong et al., 2022). Because the size of the human population and the extent of anthropogenic interference increased drastically within the range of *L. l. chinensis* after the mid‐Holocene (Dong et al., 2021), human disturbance might have been one of the major factors driving this decline in *N*_e_ and genetic diversity.

The recent decline in the Eurasian otter population might be linked to human activities since the 1950s: *N*_e_ of the Kinmen group dropped to <127 within the last four generations (28 years; Figure 2b). An unusual plateau (Ne exceeding 1400,000) occurred right before this drop. Because gene flow can cause an overestimation of *N*_e_ in GONE analysis, which can lead to such unexpected *N*_e_ (Santiago et al., 2020), this suggests that the Kinmen group might have intermingled with other otter populations before otters were wiped out in nearby areas. However, our FastSTRUCTURE and PCA evaluations showed that the individuals we studied were distinctly grouped into three groups (Figure 1). Each individual within the same group shared strong ancestral ties or was notably different from members of other groups. This indicates that the unusual plateau wasn't due to gene flow among these three groups. Instead, it signifies intermingling with an otter population we have not sampled, possibly from southern East Asia.

### 4.3 No sign of genetic health problems for the Kinmen Island population.

Small and isolated populations are expected to incur extinction risk due to the loss of genetic diversity, the accumulation of deleterious mutations, and inbreeding depression (Frankham, 2005). However, the *F*_ROH_ of otters in East Asia is not lower than that of most other carnivores sequenced (Brüniche‐Olsen et al., 2018; Figure S1) but lower than that of small and isolated carnivore populations such as the Bengal tiger (*Panthera tigris tigris*) in India (the average *F*_ROH_ = 0.57; Khan et al., 2021). Compared to critically endangered aquatic mammals, the average *F*_ROH_ of European otters is similar to that of the vaquita porpoise (*Phocoena sinus*), which has an average *F*_ROH_ = 0.05 (Robinson et al., 2022), but much lower than that of Orca *Orcinus orca* (*F*_ROH_ = 0.27, Brüniche‐Olsen et al., 2018; Kardos et al., 2023; Figure S1). The relatively low *F*_ROH_ of the Kinmen individuals (Figure S1) could result from genetic purging that had removed homologous deleterious mutations from the population through purifying selection (Hedrick & Garcia‐Dorado, 2016). However, we did not observe a lower proportion of non‐synonymous substitutions or missense substitutions, the signature of genetic purging (Robinson et al., 2022), in the Kinmen Island population. Alternatively, it might have been rendered by mating with immigrants. Large numbers of otters and a high proportion of floaters on Kinmen Island (Hung et al., 2004) hint at the presence of floaters. However, fecal DNA data (Hung et al., 2004) is not consistent with the possibility of male‐biased dispersal (Pusey, 1987) as reported in other mammals. Nevertheless, probably due to an increasing number of surveys, sightings of Eurasian otters on the southern coast of China have increased in recent years (Han & Shi, 2019). Considering the long natal dispersal distance of Eurasian otters (Quaglietta et al., 2013), numerous river systems along the southern China coast could serve as a series of stepping stones allowing otters to travel along the south China coast, including to and from the Kinmen Island. Such gene flow should introduce new genetic variants to Kinmen Island, reducing the deleterious genetic effects, such as genetic erosion, mutation load, and inbreeding.

The long‐term persistence of a small population is partly determined by whether it has sufficient genetic variations to cope with the selection pressures of an ever‐changing environment and whether the accumulated mutation load would undermine its fitness. However, we found no evidence to support genetic erosion or a high mutation load in the Kinmen Island population. However, we found no evidence to support genetic erosion or a high mutation load in the Kinmen Island population. This might be partly due to only four generations (Figure 2b) having elapsed since the drastic reduction in the otter's *N*_e,_ perhaps too short for genetic drift to have significant impacts (Kirkpatrick & Jarne, 2000). Alternatively, the lack of a signature for genetic erosion or increased mutation load might be attributed to a small amount of introgression, which would furnish some genetic variation in a small population (Madsen et al., 1999).

### 4.4 Conservation implications

At the regional scale, mitochondrial‐based phylogeny indicated that Eurasian otters in continental East Asia comprise three lineages: two for *L. l. lutra* (the Korean Peninsula and Non‐Korean Peninsula) and one for *L. l. chinesis*. However, unlike the shallow structure of the non‐Korean Peninsula clade, the *L. l. chinensis* clade comprises several deeper subclades (du Plessis et al., 2023). It supports our genomic data to treat *L. l. chinesis* into two MUs. Therefore, conservation practice should treat continental East Asian otters as at least three distinct MUs to monitor and manage independently. However, more sampling over a wide geographical area is required to discover whether more MUs exist, especially in the Korean Peninsula and southern East Asia. At a local scale, we observed that the genetic diversity of the Kinmen Island individuals is equal to that of the other more widely distributed groups. This hints that the Kinmen group could be a part of a much more widely distributed population. Therefore, connectivity between the Kinmen population and other populations within the same MU needs to be estimated with more samples from the southern coast of China. Considering its small current *N*_e_ (<50), the small surveyed population size (<100 individuals), and the unstoppable deterioration of habitats on Kinmen Island, we expect that genetic erosion, mutation load, and inbreeding depression might all become inevitable soon. Therefore, genetic rescue could become a viable management option to sustain the small Kinmen Island population. However, a lack of knowledge of the extent of the Kinmen group would hinder our ability to choose suitable source populations for any potential population supplementation projects (Bell et al., 2019). Consequently, information on the extent of the Kinmen MU would be critically important to the project's success. Our study also demonstrates the power of genomic data to unveil the details of the life history of this highly cryptic species.

Furthermore, precisely monitoring the population size of cryptic species, such as Eurasian otters, is always challenging. Non‐invasive genotyping could be a great alternative to survey otter populations precisely (Hung et al., 2004; Jang‐Liaw, 2021). However, traditional microsatellite‐based fecal genotyping methods are time‐consuming and labor‐intensive (e.g., Hung et al., 2004). It greatly restricts the utility of such techniques in otter conservation. The new genotyping technology, such as the DNA chip or array, might provide a more efficient and cost‐effective alternative for fecal DNA typing (e.g., Kraus et al., 2015). The pan‐Eurasian genetic variants generated here should give the rich resources to develop such technology for Eurasian otters to greatly enhance our ability to monitor its population.

Threatened species typically exhibit lower genetic variation (Willoughby et al., 2015). Their limited population size often results in these species living in fragmented and isolated populations. As a result, the level of ROH is expected to correlate with the IUCN Red List status. However, empirical evidence, including the current study, suggests that neither intraspecific genetic diversity (Schmidt et al., 2023) nor the level of ROH similarity reliably predicts IUCN Red List categories. Hence, relying solely on genetic characteristics to assess conservation priority for an organism could be misleading.

## CONFLICT OF INTEREST STATEMENT

The authors declare that they have no conflicts of interest.

## Supporting information


Appendix S1.

## Data Availability

Genetic data: Raw sequence reads are deposited in the SRA (BioProject PRJNA906309).
