# Supplementary material for: Low but highly geographically structured genomic diversity of East Asian Eurasian otters and its conservation implications
Source: Evol Appl. 2023 Dec 23;17(1):e13630. doi: 10.1111/eva.13630 (PMC10824276; doi:10.1111/eva.13630)
Supplement: Supplementary file 1 — Appendix S1. [file EVA-17-e13630-s001.docx]

Supplementary Figure legends

Figure S1. A. Genetic diversity (heterozygosity) , B. Mean ROH length (*L*_ROH_) and C. Genomic inbreeding coefficient, *F*_ROH_ for the three groups of Eurasian otters and published carnivorous mammal species (Brüniche-Olsen et al., 2018).

Figure S2. Results of SMC++ analysis for the A. Palearctic vs. the non-Kinmen group; B. Palearctic vs. Kinmen group, and C. Kinmen vs. non-Kinmen group. K, NK, and PA denote Kinmen, non-Kinmen, and Palearctic groups.


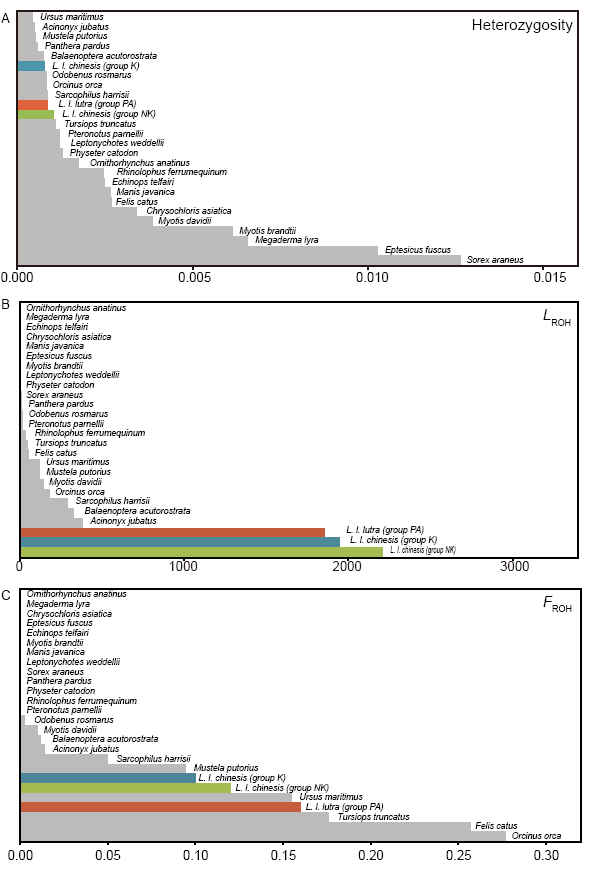


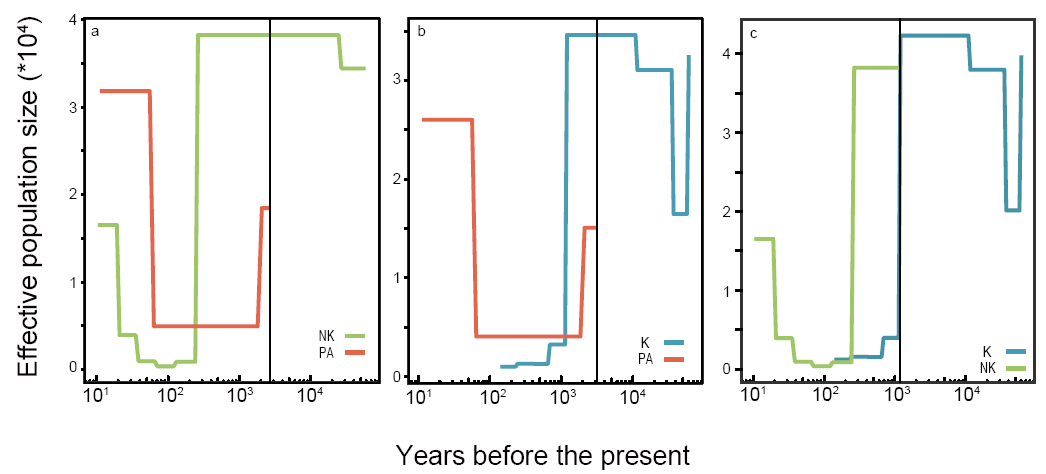


Table S1. Individual code, origin, sex, amount of raw and, filtered sequences, average sequence depth of genome for each Eurasian otter individual. UN denotes individuals had not been sexed based on morphological traits in the collection records. All Kinmen individuals collected from Kinmen Island (approximately E118.44°, N24.48°)

| Individual | Origin | Sex | Raw sequence (Gb) | Filtered sequence (Gb) | Depth (x) |
| --- | --- | --- | --- | --- | --- |
| lu_110 | German (Taipei Zoo) | Female | 176.40 | 151.74 | 50.55 |
| lu_11 | Kinmen | Male | 129.55 | 108.12 | 35.82 |
| lu_16457 | Kinmen | Female | 129.04 | 108.79 | 36.02 |
| lu_212 | Kinmen | Male | 154.72 | 129.54 | 41.66 |
| lu_3044 | Kinmen | Un | 197.82 | 133.92 | 44.04 |
| lu_3045 | Kinmen | Male | 187.77 | 156.28 | 50.65 |
| lu_408 | Kinmen | Un | 181.96 | 137.66 | 45.78 |
| lu_445 | Kinmen | Un | 178.16 | 149.07 | 48.71 |
| lu_455 | Kinmen | Female | 155.24 | 142.60 | 46.71 |
| lu_456 | Kinmen | Male | 128.38 | 106.77 | 35.46 |
| lu_5300 | Kinmen | Female | 161.33 | 135.96 | 44.00 |
| lu_7333 | Kinmen | Female | 163.40 | 141.75 | 45.70 |
| lu_7639 | Kinmen | Un | 153.79 | 126.45 | 41.29 |
| lu_86 | Kinmen | Male | 134.44 | 112.21 | 37.83 |
| lu_liu54 | Qingchuan, Sichuan, China, | Un | 154.98 | 136.00 | 55.53 |
| lu_liu55 | Qingchuan, Sichuan, China, | Un | 152.35 | 132.20 | 53.95 |
| lu_liu56 | Tahe, Heilongjiang, China | Un | 158.83 | 136.96 | 55.81 |
| lu_liu57 | Fuding, Fujian, China | Un | 154.55 | 134.58 | 54.96 |
| lu_liu58 | Xiangshan, Zhejiang, China | Un | 158.45 | 138.31 | 56.47 |

Table S2. Numbers of heterozygous autosomal SNPs, heterozygosity (*θ,* no. heterozygous autosomal SNPs/length of effective autosomal genome) for each Eurasian otter genome included in current study.

| Individual | Heterozygous SNPs | heterozygosity (10^-3^) |
| --- | --- | --- |
| *L. l. chinensis* (group K) | | |
| lu_11 | 2,134,967 | 0.914 |
| lu_16457 | 1,915,386 | 0.820 |
| lu_212 | 1,533,278 | 0.656 |
| lu_3044 | 2,062,247 | 0.883 |
| lu_3045 | 1,810,581 | 0.775 |
| lu_408 | 1,643,332 | 0.703 |
| lu_445 | 2,135,941 | 0.914 |
| lu_455 | 2,056,341 | 0.880 |
| lu_456 | 1,958,371 | 0.838 |
| lu_5300 | 1,507,672 | 0.645 |
| lu_7333 | 1,973,963 | 0.845 |
| lu_7639 | 2,034,102 | 0.871 |
| lu_86 | 2,004,434 | 0.858 |
| *L. l. chinensis* (group NK) | | |
| lu_liu57 | 1,715,628 | 0.734 |
| lu_liu58 | 2,405,944 | 1.030 |
| lu_liu54 | 1,950,766 | 0.835 |
| lu_liu55 | 2,416,311 | 1.034 |
| *L. l. lutra* (group PA) | | |
| lu_liu56 | 1,789,712 | 0.766 |
| lu_110 | 2,154,838 | 0.922 |
| mlutlut1 | 1,018,083 | 0.436 |

Table S3. Mean ROH length (*L*_ROH_), and mean number of ROHs (*N*_ROH_), and inbreeding coefficient, *F*_ROH_, of each Eurasian otter individual.

|  | *L*_ROH_ | *N*_ROH_ | *F*_ROH_ |
| --- | --- | --- | --- |
| *L. l. chinensis* (group K) | | | |
| lu_11 | 1801.11 | 104 | 0.078 |
| lu_16457 | 2038.56 | 106 | 0.090 |
| lu_212 | 2167.71 | 160 | 0.145 |
| lu_3044 | 1787.89 | 110 | 0.082 |
| lu_3045 | 2030.88 | 146 | 0.124 |
| lu_408 | 1931.1 | 172 | 0.138 |
| lu_445 | 1816.81 | 89 | 0.067 |
| lu_455 | 1854.33 | 102 | 0.079 |
| lu_456 | 1854.52 | 120 | 0.093 |
| lu_5300 | 2023.4 | 191 | 0.161 |
| lu_7333 | 1952.88 | 121 | 0.099 |
| lu_7639 | 2065.27 | 113 | 0.097 |
| lu_86 | 1952.75 | 129 | 0.105 |
| *L. l. chinensis* (group NK) | | | |
| lu_liu57 | 2811.2 | 165 | 0.193 |
| lu_liu58 | 1703.19 | 122 | 0.086 |
| lu_liu54 | 2653.92 | 149 | 0.165 |
| lu_liu55 | 1651.67 | 79 | 0.054 |
| *L. l. lutra* (group PA) | | | |
| lu_liu56 | 1624.86 | 166 | 0.112 |
| lu_110 | 1766.03 | 122 | 0.090 |
| mlutlut1 | 2184.44 | 303 | 0.273 |

Table S4 Number of non-synonymous, synonymous substitutions, stop codon gains, and stop codon losses for each Eurasian otter genome. Stop codon gains and losses are combined as loss of function (LoF) substitutions in the text.

| Individual | Nonsynonymous SNPs | Synonymous SNPs | Stop codon gain | Stop codon loss |
| --- | --- | --- | --- | --- |
| *L. l. chinensis* (group K) | | | | |
| lu_11 | 6,605 | 28,707 | 114 | 7 |
| lu_16457 | 6,388 | 28,276 | 106 | 8 |
| lu_212 | 6,038 | 27,657 | 108 | 7 |
| lu_3044 | 6,576 | 28,502 | 112 | 7 |
| lu_3045 | 6,175 | 28,007 | 99 | 3 |
| lu_408 | 5,943 | 27,376 | 117 | 6 |
| lu_445 | 6,757 | 28,603 | 112 | 8 |
| lu_455 | 6,540 | 28,320 | 109 | 8 |
| lu_456 | 6,550 | 28,412 | 111 | 9 |
| lu_5300 | 5,765 | 27,121 | 95 | 9 |
| lu_7333 | 6,354 | 28,316 | 104 | 8 |
| lu_7639 | 6,459 | 28,249 | 119 | 9 |
| lu_86 | 6,580 | 28,387 | 113 | 7 |
| *L. l. chinensis* (group NK) | | | | |
| lu_liu54 | 6,284 | 27,869 | 89 | 7 |
| lu_liu55 | 6,758 | 28,941 | 120 | 5 |
| lu_liu57 | 6,359 | 28,253 | 100 | 8 |
| lu_liu58 | 6,776 | 28,990 | 104 | 8 |
| *L. l. lutra* (group PA) | | | | |
| lu_liu56 | 5,018 | 25,888 | 87 | 7 |
| lu_110 | 5,400 | 26,212 | 95 | 10 |
| mlutlut1 | 2,009 | 20,447 | 61 | 5 |
